# The Role of Parents in Adolescent Gambling Participation and Gambling Problems

**DOI:** 10.1007/s10899-026-10520-4

**Published:** 2026-06-04

**Authors:** Cassandra K. Dittman, Mary-Cait Hunter, Michelle Gossner, Nerilee Hing, Matthew Rockloff, Matthew Browne, Lisa Lole, Alex M. T. Russell

**Affiliations:** 1 School of Health, Medical and Applied Sciences, Central Queensland University, Locked Bag 3333, Bundaberg, QLD 4670 Australia; 2 Cluster for Resilience and Wellbeing, Appleton Institute, Central Queensland University, Bundaberg, Australia; 3 School of Psychology, The University of Queensland, Brisbane, Australia; 4 Experimental Gambling Research Laboratory, Central Queensland University, Bundaberg, Australia

**Keywords:** Parenting, Parent-adolescent relationships, Adolescent gambling, Adolescent problem gambling, Gambling harm, Youth gambling

## Abstract

This study aimed to identify parental influences on adolescent gambling using an online survey of 1,183 Australian parents (69% mothers) with an adolescent aged 12–17 years. The survey assessed a comprehensive range of demographic, adolescent and parent influences on adolescent gambling participation and gambling problems, focusing on gambling-related parental factors (i.e., parent problem gambling, parental attitudes to adolescent gambling, parental facilitation of gambling), parenting practices, and the parent-adolescent relationship. The results of multiple regression analyses revealed that parental facilitation of adolescent gambling was the strongest predictor of adolescent gambling participation and gambling problems, while other significant independent predictors were adolescent simulated gambling, poor parental monitoring, and less positive parent-adolescent relationships. The results emphasize the importance of targeting parents in strategies for reducing gambling and gambling-related harm in adolescents.

**Supplementary Information:**

The online version contains supplementary material available at https://doi.org/10.1007/s10899-026-10520-4.

## Introduction

Gambling among adolescents is common (Hing et al. 2021b; King et al. 2020) despite gambling under the age of 18 years being illegal in most countries. Importantly, rates of adolescent problem gambling, characterised by a preoccupation with gambling, difficulty controlling or stopping gambling despite negative consequences, and needing to gamble more to gain the same excitement (Delfabbro & King, 2021), have been estimated to range from 1 to 7% (King et al., 2020). Parents likely play an important role in the development of adolescent gambling—including traditional and online gambling—through gambling-related behaviors and attitudes, and through broader parenting practices and relationship interactions (McComb & Sabiston, 2010). However, research on parental influences is becoming outdated (e.g., Canale et al., 2017; Molinaro et al., 2014; Vachon et al., 2004), with most research having been conducted at a time when adolescent access and exposure to gambling was considerably more limited, predating the widespread proliferation of online monetary and simulated gambling and aggressive gambling advertising (King et al., 2020). Moreover, most work has been conducted with adolescents themselves, with little research investigating adolescent gambling from the perspective of parents. This is despite research suggesting that adolescent gambling behavior is observable to parents, as it either occurs with parents or within the family home (Freund et al. 2023; Hing et al. 2021b). Therefore, contemporary research is needed with parents to identify modifiable parental risk and protective factors that can inform public health strategies and interventions to protect adolescents from gambling harm.

### Contemporary Gambling Landscape and Adolescent Gambling

This generation of adolescents is exposed to gambling in a way that no other generation has experienced. Through their frequent interactions with social networking sites, online gaming and online streaming platforms (Pew Research Center, 2024), adolescents are regularly exposed to online gambling advertising (Guillou-Landreat et al., 2021; Noble et al., 2022) and have ready access to simulated gambling activities (e.g., loot boxes within video games, social casino games; Delfabbro & King, 2021). Adolescent gambling exposure is further exacerbated in traditional broadcast media, with gambling promotions during televised sports identified as being particularly prominent (Noble et al., 2022). The impact of the widespread and intensive gambling exposure on adolescents is only just starting to be understood, with recent research showing that it is associated with favorable attitudes to gambling, poor understanding of gambling risks, future gambling intentions, and current gambling and gambling problems among adolescents (Hayer et al., 2018; Parrado-González & León-Jariego, 2020; Rossi & Nairn, 2024).

The experiences of Australian adolescents are comparable to those in other countries (Noble et al., 2022), with adolescents reporting that exposure to gambling products and marketing in their home and in the community is associated with perceptions that gambling is a normal and common activity (Royce et al., 2025; Thomas et al., 2023). Adolescent exposure to gambling sits within a broader cultural acceptance of gambling in Australia, being considered a significant aspect of Australian tradition and culture (Marko et al., 2022). Recently, gambling normalisation has been at the forefront of public and political attention in Australia due to a Federal Government enquiry into the suitablity of the current regulatory framework for online gambling and gambling advertising (Standing Committee on Social Policy and Legal Affairs, 2023). The Federal Government’s response to the enquiry, involving partial bans and ‘caps’ on the number and timing of gambling advertisements, has been criticised for failing to enact the fully evidence-based public health approach to gambling harm recommended by the enquiry (Thomas et al., 2026). What this means for Australian parents—and for many parents across the world—is that gambling continues to be brought into the family home regardless of parents’ own gambling attitudes or behavior, and that parents cannot rely on external regulatory practices (e.g., age limits for entry into gambling venues) to protect their adolescent from gambling.

### Parental Gambling Attitudes and Behavior and Adolescent Gambling

Parents are likely to be one of the strongest influences on whether the broader societal endorsement of gambling impacts the gambling behavior of adolescents (Parrado-González & León-Jariego, 2020). Within the family home, parents may express particular attitudes towards gambling or engage in certain gambling behaviors that either promote or hinder adolescent gambling participation. The most direct evidence for this notion comes from research showing that growing up with a parent experiencing problem gambling places adolescents at risk of gambling participation and gambling problems (Dowling et al. 2017a; McComb and Sabiston 2010). Further, large-scale Australian surveys of adolescents have shown that parental problem gambling uniquely predicts adolescent at-risk and/or problem gambling after controlling for key demographic factors (Dowling et al. 2017b; Hing et al. 2021b). Even within families that are not exposed to parental problem gambling, parents may express positive attitudes towards gambling that contribute to the transmission of gambling across generations. For instance, research with adolescents in the U.S. (Leeman et al., 2014; Zhai et al., 2021) and in Australia (Hing et al. 2021b) found that perceived parental approval of gambling was an independent risk factor of adolescent gambling participation.

Parental endorsement of gambling is also likely exhibited through a parent gambling in the presence of, or directly with, their adolescent child. In a study of 824 Australian high school students, a notable proportion of adolescents reported that their parents were present when they gambled on scratch tickets (20%) and bet on sports (11%; King & Delfabbro, 2016). Further, in one of the few studies with parents, Campbell et al. (2011) found that around 60% of a sample of 2,710 Canadian parents had gambled with money with their adolescents, with the most common activities being purchasing scratch tickets (40%), joint raffle tickets (36%), and joint lottery tickets (12%). In a more recent study with 1,185 Australian parents that evaluated a range of contemporary and traditional forms of gambling, 55% indicated they had gambled with their adolescent in the past 12 months, most frequently on skins betting^1^ (75%), fantasy sports betting (70%), keno (66%), and online betting (65%; Dittman et al., 2025).

Along with co-gambling, parents may endorse gambling in other ways, such as by asking their adolescent to pick “lucky numbers” in lottery or keno, involving them in selecting winning teams or score lines when online betting during televised sports games, or by talking positively about their own gambling wins. Recent qualitative research documented that adolescent gamblers were more likely than non-gamblers to recall being introduced to gambling by their parents at a young age as a part of family activities or special occasions, with adolescents reporting these as fun and positive memories (Hing et al., 2024). However, there is limited research to determine whether parental facilitation of adolescent gambling, either through co-gambling or other facilitative behaviors, has a negative impact on adolescents. Research with adolescents has shown that gambling with parents is associated with adolescent gambling participation (Hing et al. 2021b) and with adolescent gambling problems (Hsu et al., 2014). Clearly, more work is needed to examine whether parental gambling attitudes and behaviors influence adolescent gambling participation and gambling problems, particularly within the current context of easy-to-access online and simulated gambling and widespread gambling advertising.

### Broader Parental Influences on Adolescent Gambling

While parental factors specific to gambling likely play an important role in the development of adolescent gambling, the behaviors and relational processes in the broader parenting context may also play an important role (Hardoon et al., 2004; Magoon & Ingersoll, 2006). Parenting practices, including parental modelling and discipline, and parent-adolescent relationship quality are well-established predictors of adolescent disruptive and antisocial behavior problems (e.g., Hoeve et al., 2009; Pinquart, 2017). However, there has been comparatively less research examining these influences on adolescent gambling. Several studies indicate that whether parent-adolescent relationship quality hinders or promotes adolescent involvement in gambling depends on the nature of the interactions between parents and adolescents. Positive parent-adolescent relationships characterized by warmth, emotional support and connectedness have been linked with lower levels of adolescent gambling and problem gambling (Hardoon et al., 2004; Magoon & Ingersoll, 2006), with this association remaining even after controlling for other sociodemographic and individual factors (Floros et al., 2013; Molinaro et al., 2014; Pisarska & Ostaszewski, 2020). Conversely, negative parent-adolescent relationships involving hostility, conflict and intrusiveness have been associated with higher levels of adolescent gambling participation (Floros et al., 2013; Magoon & Ingersoll, 2006) and greater risk of problem gambling (Hardoon et al., 2004). Notably, in the longitudinal research by Pisarska and Ostaszewski (2020), positive relationships with parents uniquely predicted lower gambling adolescent involvement, but peer relationships did not, highlighting the central importance of parents in adolescent gambling behavior.

Research examining the role of parenting practices in adolescent gambling has largely focused on parental monitoring and parental discipline practices. Well-controlled cross-sectional studies using surveys with adolescents have shown that adolescent gambling is independently predicted by poor parental monitoring (Canale et al., 2017; Molinaro et al., 2014; Vachon et al., 2004) and ineffective discipline (Vachon et al., 2004). Longitudinal research has produced mixed findings. Two Canadian studies found that adolescent-reported parental monitoring during early or mid-adolescence did not uniquely predict gambling participation or problem gambling later in adolescence (Vitaro et al., 2001; Wanner et al., 2009). In contrast, a U.S. study found that adolescents who reported low and declining levels of parental monitoring between 11 and 14 years were significantly more likely to report problem gambling by age 22 in comparison to young people who experienced high and stable levels of parental monitoring (Lee et al., 2014).

It is important to note that most of this research was conducted over a decade ago. Updated research is needed to determine whether these findings apply to contemporary adolescents, given the vast technological and social changes since that time, including in relation to gambling (King et al., 2020). The nature of parental monitoring and supervision has also altered in response to these changes. Increased accessibility to technology and the internet has meant that parental monitoring and limit-setting now needs to consider young people’s online activities and usage of smartphones, tablets, and other internet-connected devices (Padilla-Walker et al., 2018; Vaala & Bleakley, 2015). This expansion of parents’ monitoring may be particularly pertinent to gambling. Through smartphone apps, social media sites and online video games, adolescents now have ready access to a vast array of online gambling activities (e.g., sports betting, online casinos, virtual poker; King et al., 2020), and simulated gambling activities (Hing et al., 2022). Research on the impact of parental monitoring and limit-setting of online activities on adolescent gambling has begun to emerge in the past decade, with a study with Greek adolescents (Floros et al., 2013) and one with Australian adolescents (King & Delfabbro, 2016) indicating that parental online monitoring is not related to adolescent gambling. Again, however, even this research needs updating given the constantly changing online gambling landscape.

### Hypotheses

This study involved an online survey of mothers and fathers of adolescents (aged 12 to 17 years) to identify the parental factors specific to gambling, along with those within the broader parenting context, that are associated with adolescent gambling. The overall aim was to provide a comprehensive examination of parental influences on adolescent gambling participation and problems. Given potential differences between maternal and paternal influences on adolescent behavior (Dowling et al. 2017b), the following hypotheses were examined separately for mothers and fathers. Hypothesis 1 predicted that parental factors specific to gambling would be associated with greater adolescent gambling participation and gambling problems. Specifically, we hypothesized that (a) more positive parental gambling attitudes, (b) higher parent problem gambling, and (c) greater parental facilitation of gambling would be related to higher levels of adolescent gambling. Hypothesis 2 predicted that broader parenting factors would also be related to greater adolescent gambling. It was predicted that (a) less positivity and more negativity in the parent-adolescent relationship, (b) lower parental monitoring (generally and for online behavior), and (c) higher use of ineffective discipline practices would be related to greater adolescent gambling.

Finally, the study aimed to identify which parental influences make an independent contribution to adolescent gambling after controlling for parental demographics (i.e., age, education, Indigenous status, household income, family structure) and adolescent factors (i.e., gender, impulsivity, mental health problems, simulated gambling) that have been implicated in adolescent gambling (Dowling et al., 2017; Hing et al., 2022). No directional hypotheses were specified for this component due to the lack of strong theoretical guidance.

## Method

### Participants and Recruitment

Participants were 1,183 parents or guardians of at least one adolescent aged 12 to 17 years living in New South Wales, Australia (the location of the funding body). Participants were recruited in June 2022 via Qualtrics, an online market research panel aggregator. Given fathers are often difficult to engage in parenting research (Fabiano & Caserta, 2018), a quota was set to attract a sufficient sample of male respondents to enable separate analyses by parent gender. If parents had more than one child in their household aged between 12 and 17 years, they were instructed to choose the child with the next birthday as the target adolescent. This method of within-household respondent selection is routinely used in population surveys and provided a non-intrusive and quasi-random method of choosing a target adolescent for survey completion (Yan, 2009).

Qualtrics recorded 1,415 responses to the survey. Several participants who reported that they were the biological parent of the target adolescent were likely too young to be a biological parent of a child that age (i.e., < 15 years difference in age). Thus, responses were removed if there was less than 15 years difference and the reported relationship did not plausibly indicate either biological parenthood or a legitimate guardian role (e.g., sibling or peer relationships without evidence of guardianship). Participant responses were then assessed for data quality, with 230 responses removed for one or more of the following reasons: duplicate responses (203); being too young to be a biological parent of an adolescent (48); poor quality responses, such as irrelevant text responses or speeding through the survey (38); having an IP address outside of Australia (17) or postcode outside of NSW (2); straight-lining (selecting the same answer through some or all of the survey when inappropriate to do so (11); and/or the survey being incomplete (10).

### Procedure

The study was approved by the authors’ university Human Research Ethics Committee (approval no. 0023502). Participants were reimbursed with a non-monetary incentive for their participation by their online survey panel provider. The informed consent process and survey completion was conducted online. Non-consenting participants were screened out of the survey. Those who did consent responded to four screening questions to assess the study inclusion criteria. Participants were screened out if they indicated: (1) they were not a resident of NSW or provided a non-NSW postcode; (2) they were not a parent or guardian of a child aged between 12 and 17 years; or (3) they responded “zero” when asked how many children were in their household. Eligible participants were then directed to the online survey. Median completion time was 19 min.

### Measures

#### Demographics

Participants provided demographic information about themselves and their adolescent. This included the participant’s age, gender, relationship status, education level, employment status, yearly household income, Indigenous status, primary language spoken at home, family structure, and relationship to the adolescent. Participants also reported their adolescent’s year of birth and gender.

##### **Adolescent Gambling Participation**

Fourteen gambling activities as specified in population surveys of adult gambling (Browne et al. 2019a, b) and adolescent gambling (Hing et al. 2021b) were used to assess parental knowledge of their adolescent’s gambling behavior. Parents reported on their adolescent’s involvement in traditional (e.g., sports betting, lottery, pokies) and modern forms of gambling (e.g., eSports betting, online casino games, skins betting). All activities in the survey are illegal for people aged under 18 years in Australia, except for informal betting with family and friends. Parents indicated “yes”, “no”, or “don’t know” as to whether their adolescent had participated in each activity for money in the past 12 months. A total participation score was calculated by summing the number of gambling activities reported during the past 12 months.

##### **Adolescent Problem Gambling**

To assess adolescent problem gambling, the survey used the Diagnostic Statistical Manual of Mental Disorders-IV-Multiple Response Format for Juveniles (DSM-IV-MR-J; Fisher, 2000) adapted for parent report. The DSM-IV-MR-J is a well-established measure frequently used in population prevalence studies of adolescent gambling (Delfabbro & King, 2021) chosen because it aligns with diagnostic criteria for problematic gambling and has adequate psychometric characteristics in comparison to other available measures (Edgren et al., 2016). This scale contains 12 items corresponding to nine diagnostic criteria for gambling disorders. The self-report items on the DSM-IV-MR-J were reworded to be suitable for parent report. For instance, the item, “Have you ever gambled to help escape from problems when you are feeling bad?” became “How often has your teenager gambled to help them escape from problems when they are feeling bad?” The rating scale was also adapted. Along with the 4-point Likert scale used in the self-report adolescent version (i.e., 0 (*never*) to 4 (*often*), the option to respond “I don’t know” was added to capture the possibility that parents were not aware of a particular problematic adolescent gambling behavior. Scoring was conducted as per the adolescent-report scale, with a total score obtained by summing the number of diagnostic criteria endorsed by the parent (α = 0.93).

#### Parental Gambling Attitudes and Behaviors

##### **Parental Attitudes to Gambling**

Parental attitudes towards adolescent gambling were assessed using three items taken from previous surveys of adolescent gambling; two items from a Canadian study (i.e., “There is nothing wrong with teens gambling occasionally” and “It is acceptable for teenagers to watch professional tournaments or TV shows featuring gambling;” Campbell et al., 2011) and one item from an Australian study (i.e., “It is OK for teenagers to play gambling games online or on a phone (e.g., casino games, pokie machines) as long as it is not for money;” Hing et al. 2021b). Each item was rated on a 5-point scale ranging from 1 (*strongly disagree*) to 5 (*strongly agree).* A mean total score was created by averaging scores across the three items; this score had adequare internal consistency, α = 0.81.

##### **Parental Problem Gambling**

The Problem Gambling Severity Index (PGSI; Ferris & Wynne, 2001) assessed parental problem gambling. Participants indicated the frequency of their participation in nine gambling behaviors (e.g., “Have you bet more than you could really afford to lose?”) in the past 12 months on a 4-point Likert scale ranging from 0 (*never*) to 3 (*almost always*). Total scores were computed by summing item scores, with higher scores indicating greater problem gambling severity (α = 0.96).

##### **Parental Facilitation of Adolescent Gambling**

Items to assess parental behaviors that facilitate gambling were included based on previous research exploring adolescent reports of parental gambling-related behavior (Hing et al. 2021b; King and Delfabbro 2016) and the Canadian parent survey (Campbell et al., 2011). Parents rated how frequently within the past 12 months they had engaged in 14 behaviors with their adolescent, including behaviors that related to involving their adolescent in gambling (e.g., “Put a bet on for them for a sport, racing, novelty or esports event”) and those that related to endorsing gambling (e.g., “Talked about your own gambling wins”). The list of parental behaviors and their frequency of endorsement by parents can be found in Supplementary Material 1. Respondents rated each behavior on a scale from 0 (*never in the past 12 months*) to 6 (*4 or more times per week*).

Principal Components Analyses (PCA) were conducted on the set of 14 items to assess suitability of combining scores on these items into a scale score for subsequent analyses. Based on inspection of the scree plot and examination of eigenvalues above 1, the PCA indicated that a one-factor model best fit the items, accounting for 79.68% of variance in scores. Item factor loadings ranged from 0.82 to 0.94, and internal consistency was strong, α = 0.98. Thus, participant scores on these items were averaged to create a total mean score, with higher scores indicating greater parental facilitation of adolescent gambling.

#### Parenting Practices and Relationships

##### **Parenting Practices**

Parenting practices were assessed using the short form of the Alabama Parenting Questionnaire (APQ; Elgar et al., 2007). The APQ short form comprises three subscales each containing three items: Positive Parenting (e.g., “You praise your teenager if they behave well”), Inconsistent Discipline (e.g., “You threaten to punish your teenager and then do not actually punish them”), and Poor Supervision (e.g., “Your teenager goes out with friends you don’t know”). The Positive Parenting and Inconsistent Discipline subscales were used to assess use of effective and ineffective discipline practices, while the Poor Supervision subscale assessed parental monitoring. Participants rated how frequently they used each parenting practice on a 5-point Likert scale ranging from 1 (*never or almost never*) to 5 (*almost always or always*). Subscale scores were calculated by averaging item responses, with higher scores indicating greater use of the respective parenting strategy. Each subscale had adequate internal consistency: α = 0.85 for Positive Parenting; α = 0.72 for Inconsistent Discipline; α = 0.83 for Poor Supervision.

##### **Parent-adolescent Relationship**

The Connectedness and Hostility subscales from the Parent-Adolescent Relationship Scale (Burke et al., 2021) assessed positivity and negativity in the parent-adolescent relationship, respectively. Participants reported on a 6-point Likert scale from 0 (*not at all true*) to 5 (*nearly always/always true*) how much each statement represented their relationship with their adolescent. The 6-item Connectedness subscale (e.g., *I encourage my teenager to get support from me or others*) demonstrated strong internal consistency in the current sample (α = 0.91) as did the 5-item Hostility subscale (α = 0.85; e.g., *I criticize my teenager*). A total mean score was created for each subscale, and higher scores reflected greater levels of that dimension of the parent-adolescent relationship.

##### Parental Online Monitoring

Six items were constructed to assess parental monitoring and regulation of their adolescent’s online activities. Items were developed based on research on parental media monitoring (Khurana et al., 2015; Padilla-Walker et al., 2018) and via consultation with the funder of this research, a state-based responsible gambling organisation. Participants rated how often they engaged in each behavior on a 5-point Likert scale from 1 (*Never or almost never)* to 5 (*Almost always or always*).

PCA with oblique (oblimin) rotation on the set of online monitoring items indicated that a two-factor model best fit the data, with the first component explaining 52.36% of variance and the second explaining 18.88% of variance. The four items on the first factor had factor loadings between 0.73 and 0.90 and related to parents setting limits and restrictions on adolescent’s online use (e.g., “You restrict or block certain websites that your teenager might use”; “You set limits about the amount of time your teenager spends online”). This subscale was therefore labelled “online restrictions”. The second factor had two items, “Your teenager spontaneously talks to you about what they are doing or seeing online” (factor loading = 0.94) and “You talk to your teenager about what they are doing online” (factor loading = 0.77). This subscale was labelled “online conversations”. The internal consistency for both subscales was adequate: α = 0.83 for online restrictions; and α = 0.69 for online conversations. The online conversations subscale was limited by the fact it contained only two items. However, the high correlations between the two items and the strong alignment in content warranted combining them into a single variable. Thus, a mean total score was created for each of these aspects of online monitoring, with higher scores corresponding to higher parental monitoring.

#### Adolescent Influences

##### **Adolescent Mental Health**

Adolescent mental health was assessed using the Emotional Difficulties subscale from the Adolescent Functioning Scale (AFS; Dittman et al., 2016), which contains five items assessing symptoms of depression and anxiety in adolescents (e.g., “Seems unhappy or sad”). Responses were rated on a 5-point Likert scale ranging from 0 (*not at all true*) to 5 (*true most of the time*) in relation to the target adolescent in the past 4 weeks. Scores on the items were averaged to yield a mean total score (α = 0.88).

##### **Adolescent Impulsivity**

The Barratt Impulsiveness Scale-Brief (BIS; Steinberg et al., 2013) assessed parental perspectives of adolescent impulsivity. The eight items on the BIS were reworded for parent report; for instance, the item, “I plan tasks carefully” was changed to, “My teenager plans tasks carefully.” Respondents indicated how frequently each statement described their adolescent from 1 (*rarely or never*) to 4 (*almost always or always*). A total score was calculated, with higher scores indicating greater adolescent impulsivity (α = 0.85).

##### **Adolescent Simulated Gambling**

Parents were asked about their adolescent’s participation in four simulated gambling activities: playing video games with gambling components, purchasing loot boxes with real money, playing gambling-like games (e.g., simulated pokies, poker, roulette), and playing free demo or practice games on gambling websites. These items were taken from a prior Australian survey on adolescent gambling (Hing et al. 2021b). Participants reported how often their adolescent took part in each activity in the past 12 months, from 0 (*never in the past 12 months*) to 6 (*4 or more times per week*). Participants were also given the option to respond “I don’t know” to each item. The number of activities the adolescent had taken part in at least once during the past 12 months was summed to create a total score (α = 0.83).

### Data Analyses

All analyses were conducted using IBM SPSS Statistics for Windows version 28. There were no missing data because all survey questions were compulsory. Bivariate correlations identified the demographic, adolescent, and parental correlates of adolescent gambling. Multiple regression analyses were then used to identify the factors that uniquely predict adolescent gambling participation and adolescent problem gambling. Separate models were developed for mothers and fathers to identify maternal versus paternal influences. Four models were evaluated for each dependent variable: (1) sociodemographic factors; (2) adolescent factors (age; gender; mental health; impulsivity; simulated gambling participation); (3) parental influences (parent problem gambling, parent attitudes to adolescent gambling, parental facilitation of adolescent gambling, parenting practices, parent-adolescent relationship factors); and (4) sociodemographic factors, adolescent factors and parental influences together. Sociodemographic factors and parental influences were modelled separately to recognize that proximal risk factors (e.g., parent gambling problems) are likely mediators of distal risk factors (e.g., parent demographics). Models that include both proximal and distal risk factors that likely have complex interrelationships are technically a model misspecification (Browne et al. 2019a, b). Thus, approaching them separately enabled a more straightforward interpretation of unique effects. Given the number of predictors examined, interpretation focuses on effects that were consistent across bivariate and multivariate analyses and across parent gender, with smaller or isolated effects interpreted cautiously.

## Results

### Sample Demographic Characteristics

Parents ranged in age from 20 to 73 years (*M* = 42.55 years, *SD* = 6.84 years)^2^. Most participants (98.3%) indicated that they were the biological or adoptive parent (94.9%) or stepparent (3.4%) of their target adolescent (see Table 1 for participant characteristics). Around two-thirds of respondents were mothers (68.9%). Most parents were married or in a cohabiting relationship (80.3%), spoke English as their main language at home (92.8%) and had a post-school qualification (e.g., trade qualification or university degree; 82.7%). Most were employed full- or part-time (84.2%), with around half (56.7%) reporting an annual income above $90,000 (cf., median annual household income of $92,872 in Australia in 2019-20; Australian Bureau of Statistics, 2022). A portion of respondents (7.2%) identified as Indigenous (cf., 3.8% in the Australian adult population; ABS, 2023). Target adolescents ranged in age from 12 to 17 years (*M* = 14.65 years, *SD* = 1.69 years). Parents responded about slightly more males (53.8%) than females (45.7%). Six parents reported their adolescent identified as a gender other than male or female. Two-thirds of adolescents lived in two-parent households (66.3%), with 19.7% living in a sole-parent household.

**Table 1:** Summary of demographic characteristics for parents (*N* = 1,183)

	*n*	%
Parent gender		
Male	367	31.0
Female	816	69.0
Adolescent gender		
Male	636	53.8
Female	541	45.7
Other	6	0.5
Parent relationship to adolescent		
Biological or adoptive parent	1124	95.0
Stepparent	40	3.4
Other (e.g., grandparent, aunt, guardian)	19	1.6
Family composition		
Two-parent original family	789	66.7
Stepfamily (two parents, one being a stepparent)	105	8.9
Blended family	55	4.6
Sole parent family	234	19.8
Marital status		
Married or living with a partner	950	80.3
Separated or divorced (not living with a partner)	156	13.2
Single/never married/never cohabiting relationship	62	5.2
Other	15	1.2
Employment status		
Working full-time	700	59.2
Working part-time	295	24.9
Home duties	136	11.5
Retired/pensioner	21	1.8
Unemployed and looking for work	22	1.9
Other	9	0.7
Highest educational attainment		
Less than a secondary school education	93	7.9
Completed secondary school	113	9.6
A trade, technical certificate, or diploma	399	33.7
Undergraduate university degree	361	30.5
Postgraduate university degree	217	18.3
Annual household income		
Less than $30,000	69	5.8
$30,001 to $50,000	90	7.6
$50,001 to $70,000	152	12.9
$70,001 to $90,001	140	11.8
$90,001 to $120,000	234	19.8
$120,001 to $160,000	229	19.4
$160,001 to $200,000	122	10.3
$200,001 and above	89	7.5
Did not wish to disclose	58	4.9
Indigenous status		
Indigenous	85	7.2
Not Indigenous	1098	92.8
Main language spoken at home		
English	1097	92.7
Language other than English	86	7.3

### Bivariate Correlates of Adolescent Gambling

Descriptive statistics and correlations between predictor and criterion variables are reported separately for mothers and fathers in Table 2 (correlations among all predictor and criterion variables are reported in Supplementary Material 2). In terms of parental demographic factors, younger parent age and being Indigenous were positively correlated with adolescent gambling participation and gambling problems for both mothers and fathers. Higher parental education was a weak positive correlate of mother-reported gambling problems and father-reported gambling participation and problems. Family income, family structure and being from a non-English-speaking background did not significantly correlate with adolescent gambling in either sample.

**Table 2:** Bivariate relationships (95% confidence intervals) between demographic, adolescent and parental factors and adolescent gambling

	Mothers (*N* = 816)			Fathers (*N* = 367)		
	M (SD)	Adolescent gambling participation *r*	Adolescent gambling problems *r*	M (SD)	Adolescent gambling participation *r*	Adolescent gambling problems *r*
Parent demographic factors						
Age (years)	41.99 (6.61)	− 0.115*** (-0.182, − 0.047)	− 0.110** (-0.177, − 0.042)	43.72 (7.07)	− 0.164** (-0.262, − 0.062)	− 0.188*** (-0.285, − 0.087)
Indigenous status (ref: not)	--	0.175*** (0.108, 0.241)	0.173*** (0.106, 0.239)	--	0.220*** (0.120, 0.315)	0.238*** (0.139, 0.332)
English as main language (ref: no)	--	0.016 (-0.052, 0.085)	0.023 (-0.046, 0.092)	--	0.063 (-0.040, 0.164)	0.000 (-0.102, 0.103)
Education	--	0.034 (-0.035, 0.102)	0.127*** (0.059, 0.194)	--	0.174** (0.073, 0.272)	0.170*** (0.069, 0.268)
Income^a^	--	0.053 (-0.018, 0.124)	0.032 (-0.068, 0.069)	--	0.028 (-0.076, 0.131)	0.046 (-0.058, 0.149)
Family structure	--	− 0.008 (-0.076, 0.061)	0.000 (-0.068, 0.069)	--	− 0.056 (-0.157, 0.047)	− 0.042 (-0.143, 0.061)
Adolescent factors						
Age (years)	14.69 (1.72)	0.011 (-0.057, 0.080)	− 0.055 (-0.123, 0.014)	14.56 (1.63)	− 0.017 (-0.119, 0.086)	− 0.061 (-0.163, 0.041)
Gender (ref: male)	--	− 0.024 (-0.093, 0.045)	− 0.013 (-0.082, 0.056)		− 0.124* (-0.224, − 0.022)	− 0.132* (-0.231, − 0.030)
Impulsivity	18.53 (4.72)	0.025 (-0.044, 0.093)	0.040 (-0.028, 0.109)	17.83 (4.11)	0.107* (0.005, 0.207)	0.142*** (0.040, 0.241)
Mental health problems	1.79 (1.13)	0.160*** (0.093, 0.227)	0.252*** (0.187, 0.315)	1.67 (1.20)	0.415*** (0.327, 0.496)	0.526*** (0.447, 0.596)
Simulated gambling participation (past 12 months)	0.38 (0.88)	0.540*** (0.489, 0.587)	0.575*** (0.527, 0.619)	1.05 (1.49)	0.590*** (0.519, 0.653)	0.720*** (0.667, 0.766)
Parental factors – general						
Positive parenting	4.23 (0.69)	− 0.187*** (-0.253, − 0.120)	− 0.235*** (-0.298, − 0.169)	3.93 (0.73)	− 0.248*** (-0.342, − 0.149)	− 0.175*** (-0.273, − 0.074)
Inconsistent discipline	2.34 (0.78)	0.179*** (0.112, 0.245)	0.205*** (0.138, 0.270)	2.50 (0.86)	0.311*** (0.216, 0.401)	0.384*** (0.293, 0.468)
Poor supervision	1.75 (0.83)	0.317*** (0.254, 0.377)	0.375*** (0.314, 0.432)	2.22 (0.96)	0.461*** (0.376, 0.538)	0.513*** (0.433, 0.584)
Online restrictions (monitoring)	2.65 (1.03)	0.076* (0.007, 0.144)	0.089* (0.020, 0.157)	2.73 (0.98)	0.264*** (0.167, 0.357)	0.318*** (0.223, 0.407)
Online conversations (monitoring)	3.44 (0.87)	− 0.078* (-0.146, − 0.010)	− 0.128*** (-0.195, − 0.060)	3.16 (0.82)	0.150** (0.048, 0.248)	0.196*** (0.096, 0.293)
Parent-adolescent connectedness	4.07 (0.92)	− 0.209*** (-0.274, − 0.142)	− 0.282*** (-0.344, − 0.218)	3.49 (0.94)	− 0.218*** (-0.313, − 0.118)	− 0.295*** (-0.386, − 0.198)
Parent-adolescent hostility	1.49 (0.90)	0.193*** (0.126, 0.258)	0.239*** (0.174, 0.303)	1.67 (1.09)	0.451*** (0.365, 0.529)	0.476*** (0.393, 0.552)
Parental factors – gambling-related						
Parent problem gambling	1.55 (3.88)	0.428*** (0.370, 0.483)	0.522*** (0.417, 0.571)	4.27 (6.18)	0.567*** (0.493, 0.632)	0.714*** (0.659, 0.760)
Parent attitudes to adolescent gambling	1.88 (0.80)	0.356*** (0.294, 0.414)	0.325*** (0.262, 0.385)	2.27 (1.05)	0.444*** (0.358, 0.522)	0.529*** (0.451, 0.599)
Parental facilitation of adolescent gambling	0.24 (0.64)	0.657*** (0.616, 0.694)	0.633*** (0.590, 0.672)	0.76 (1.30)	0.716*** (0.662, 0.763)	0.797*** (0.757, 0.832)
Adolescent gambling participation	0.57 (1.58)	--	0.575*** (0.528, 0.619)	1.73 (3.32)	--	0.625*** (0.558, 0.684)
Adolescent gambling problems	0.37 (1.06)	0.575*** (0.528, 0.619)	--	1.29 (2.35)	0.625*** (0.558, 0.684)	--

Note. Both parametric (i.e., Pearson’s *r*) and non-parametric (i.e., Spearman’s Rho) correlations were calculated for categorical predictor variables. Because there was no substantive difference in the results of these analyses, Pearson’s *r* values are reported

^a^ Based on *N* = 767 for mothers and *N* = 358 due to respondents indicating they did not know or wish to disclose their family income

* *p* < .05, ** *p* < .01, *** *p* < .001

Regarding adolescent factors, higher levels of adolescent mental health problems and higher participation in simulated gambling were related to greater adolescent gambling participation and problems in both the mother and father samples. In the father sample only, adolescent male gender and higher impulsivity were correlates of adolescent gambling. Adolescent age was not related to adolescent gambling in either sample.

Most general parental factors were weakly to moderately associated with adolescent gambling. Mothers and fathers who reported lower positive parenting, higher inconsistent discipline, and poorer supervision also reported higher adolescent gambling participation and problems. Both forms of online monitoring were correlates, although only weakly for mothers. Higher online restrictions were related to both measures of adolescent gambling for mothers and fathers. However, for fathers, more online conversations were associated with adolescent gambling, while fewer online conversations were associated with adolescent gambling for mothers. In terms of parent-adolescent relationship quality, mothers and fathers who reported higher levels of hostility and lower connectedness with their adolescent also reported higher adolescent gambling.

Finally, gambling-related parental influences were moderately to strongly related to adolescent gambling for both mothers and fathers. Higher levels of parent problem gambling, more positive attitudes to adolescent gambling, and greater parental facilitation of gambling were related to adolescent participation in gambling and gambling problems.

### Multiple Regression Analyses

Assessment of the assumptions of linear regression revealed no violation of the assumptions of homoscedasticity, and independence of observations and there were no influential cases at a multivariate level. Because of the strong correlations among several of the independent variables (see Supplementary Material 2), multicollinearity was assessed via inspection of Tolerance and Variance Inflation Factor (VIF) values with no violations of this assumption identified. Specifically, the results revealed that VIF values were within acceptable limits, being < 3 in the mothers’ (values ranged from 1.25 to 2.66) and < 4 in the fathers’ data (values ranged from 1.36 to 3.58). Similarly, Tolerance values were acceptable, being > 0.2 in both the mothers’ (ranged from 0.38 to 0.85) and fathers’ samples (ranged from 0.28 to 0.74).

Checks for violations of normality revealed that, in both the mother and father samples, the distributions of the scores on the DSM-IV-MR-J, adolescent gambling participation, PGSI, and parental facilitation of gambling were positively skewed. There were also several univariate outliers in each of these distributions. When logarithmic transformations were conducted to reduce the influence of the skew and outliers, there were no substantive changes to the outcome of the correlational analyses or the multiple regression analyses. Thus, the original, non-transformed scores were retained in regression analyses.

Given the large number of predictors examined, there is potential for inflation of Type I error. The primary inferences of this study are therefore based on predictors that demonstrated consistent effects across bivariate and multivariate analyses, across outcomes, and across mother and father reports. Findings involving smaller or isolated effects should be interpreted cautiously and considered exploratory.

### Factors Influencing Adolescent Gambling Participation

Variables significant at the bivariate level were entered into multivariate regression models to assess the contribution of demographic, adolescent and parental influences on adolescent gambling participation. The first regression model assessed the influence of parental demographic factors on adolescent gambling participation. For mothers (see Table 3) and fathers (see Table 4), younger parent age and Indigenous status were significant predictors, while higher parent education was also significant in the model for fathers. The second model examined the association between adolescent factors and gambling participation. In both the mother and father models, higher adolescent emotional problems and 12-month participation in simulated gambling were significant predictors. Adolescent gender and impulsivity were also entered in the model for fathers, since both were significant bivariate correlates in analyses with the father sample. However, neither of these variables explained unique variance in this multivariate model. The third model examined the independent influence of broader parental influences and gambling-related parental influences on adolescent gambling participation. In the analysis of mother data, poor supervision, positive parent attitudes to adolescent gambling and parental facilitation of gambling were unique predictors. In the analysis with fathers, lower positive parenting, greater parent-adolescent hostility, and greater parental facilitation of gambling were significant predictors.

**Table 3:** Results of linear regression analyses with mothers (n = 816) examining predictors of adolescent gambling participation

	Model 1: Parent demographic factors			Model 2: Adolescent factors			Model 3: Parental factors			Model 4: All predictors simultaneously		
	B	SE	β	B	SE	β	B	SE	β	B	SE	β
Parent age	− 0.022	0.008	− 0.091*							0.001	0.007	0.004
Indigenous status (ref: not)	1.028	0.221	0.161***							0.419	0.173	0.066*
Adolescent mental health problems				0.087	0.042	0.062*				− 0.015	0.040	− 0.011
Adolescent simulated gambling				0.950	0.054	0.528***				0.227	0.067	0.126***
Positive parenting							− 0.009	0.026	− 0.012	− 0.010	0.025	− 0.013
Inconsistent discipline							0.006	0.021	0.008	0.007	0.020	0.011
Poor supervision							0.048	0.020	0.076*	0.044	0.020	0.070*
Online restrictions (monitoring)							0.015	0.045	0.010	0.028	0.046	0.018
Online conversations (monitoring)							0.088	0.059	0.048	0.069	0.059	0.038
Parent-adolescent connectedness							− 0.053	0.062	− 0.031	− 0.033	0.062	− 0.019
Parent-adolescent hostility							− 0.041	0.054	− 0.023	− 0.043	0.055	− 0.025
Parent problem gambling							0.012	0.014	0.029	0.006	0.014	0.014
Parent attitudes to teen gambling							0.168	0.058	0.086**	0.154	0.058	0.078**
Parental facilitation of gambling							1.432	0.090	0.576***	1.234	0.106	0.496***
	*R*^2^ = 0.039, *F*(2, 813) = 16.361, *p* < .001			*R*^2^ = 0.295, *F*(2, 813) = 170.333, *p* < .001			*R*^2^ = 0.448, *F*(10, 805) = 65.310, *p* < .001			*R*^2^ = 0.462, *F*(14, 801) = 49.062, *p* < .001		

Note. * *p* < .05, ** *p* < .01, *** *p* < .001

**Table 4:** Results of linear regression analyses with fathers (*N* = 367) examining predictors of adolescent gambling participation

	Model 1: Parent demographic factors			Model 2: Adolescent factors			Model 3: Parental factors			Model 4: All predictors simultaneously		
	B	SE	β	B	SE	β	B	SE	β	B	SE	β
Parent age	-0.058	.024	-0.123*							0.012	0.018	0.025
Indigenous status (ref: not)	2.399	605	0.201***							0.880	.0457	0.074
Parent education	0.569	0.164	0.173***							0.078	0.123	0.024
Adolescent gender (ref: male)				− 0.322	0.294	− 0.047				− 0.035	0.258	− 0.005
Adolescent impulsivity				− 0.025	0.037	− 0.031				− 0.021	0.035	− 0.027
Adolescent mental health problems				0.552	0.141	0.200***				− 0.126	0.148	− 0.046
Adolescent simulated gambling				1.093	0.105	0.493***				0.171	0.129	0.077
Positive parenting							− 0.164	0.072	− 0.107*	− 0.198	0.073	− 0.130**
Inconsistent discipline							− 0.053	0.062	− 0.041	− 0.043	0.067	− 0.033
Poor supervision							0.039	0.063	0.033	0.031	0.063	0.027
Online restrictions (monitoring)							0.037	0.151	0.011	0.040	0.153	0.012
Online conversations (monitoring)							0.189	0.179	0.047	0.207	0.183	0.052
Parent-adolescent connectedness							0.054	0.165	0.015	0.044	0.168	0.013
Parent-adolescent hostility							0.328	0.159	0.100*	0.373	0.163	0.114*
Parent problem gambling							0.022	0.030	0.041	0.006	0.031	0.011
Parent attitudes to teen gambling							0.058	0.144	0.018	0.033	0.144	0.010
Parental facilitation of gambling							1.528	0.152	0.596***	1.436	0.175	0.564***
	*R*^2^ = 0.095, *F*(3, 363) = 12.628, *p* < .001			*R*^2^ = 0.374, *F*(4, 361) = 54.029, *p* < .001			*R*^2^ = 0.538, *F*(10, 356) = 41.488, *p* < .001			*R*^2^ = 0.551, *F*(17, 348) = 25.108, *p* < .001		

Note. * *p* < .05, ** *p* < .01, *** *p* < .001

The final model assessed demographic factors, adolescent factors and parental influences simultaneously. In the analysis with mothers, Indigenous background, simulated gambling participation, poor supervision, positive parental gambling attitudes and parental facilitation of gambling each contributed independent variance to adolescent gambling participation. For fathers, the unique predictors were lower positive parenting, greater parent-adolescent hostility and greater parental facilitation of gambling. Parental facilitation of gambling was the strongest predictor for mothers and fathers.

### Factors Influencing Adolescent Gambling Problems

These analyses address the hypotheses concerning gambling-specific parental factors and broader parenting influences on adolescent gambling participation. In the first regression model involving parent demographic factors, parent age, Indigenous status and parental education each uniquely predicted adolescent gambling problems in the mother (see Table 5) and father samples (see Table 6). The second model assessed the influence of adolescent factors. For mothers and fathers, the unique predictors were adolescent mental health problems and simulated gambling participation. Model 2 for fathers also included adolescent gender and impulsivity, but neither of these were significant predictors. Results of the third regression model, which evaluated parental influences on adolescent gambling problems, indicated that the independent predictors in the mother sample were poor supervision, parent-adolescent connectedness, parent problem gambling and parental facilitation of gambling. In the analysis of father data, parent-adolescent connectedness, parent problem gambling and parental facilitation of gambling were also significant, but poor supervision was not. However, positive parenting was a significant predictor for fathers.

**Table 5:** Results of linear regression analyses with mothers (*N* = 816) examining predictors of adolescent gambling problems

	Model 1: Parent demographic factors			Model 2: Adolescent factors			Model 3: Parental factors			Model 4 All predictors simultaneously		
	B	SE	β	B	SE	β	B	SE	β	B	SE	β
Parent age	-0.014	0.006	-0.086*							0.005	0.004	0.034
Indigenous status (ref: not)	0.719	0.147	0.169***							0.150	0.112	0.035
Parent education	0.128	0.032	0.139***							0.086	0.024	0.093***
Adolescent mental health problems				0.141	0.027	0.150***				0.061	0.026	0.065*
Adolescent simulated gambling				0.657	0.035	0.547***				0.270	0.043	0.225***
Positive parenting							− 0.009	0.017	− 0.017	0.000	0.016	− 0.001
Inconsistent discipline							− 0.006	0.014	− 0.014	− 0.006	0.013	− 0.014
Poor supervision							0.052	0.013	0.123***	0.045	0.013	0.105***
Online restrictions (monitoring)							0.054	0.030	0.053	0.058	0.030	0.057
Online conversations (monitoring)							0.020	0.039	0.016	0.008	0.038	0.006
Parent-adolescent connectedness							− 0.097	0.041	− 0.084*	− 0.106	0.040	− 0.092**
Parent-adolescent hostility							− 0.009	0.036	− 0.007	− 0.031	0.035	− 0.026
Parent problem gambling							0.052	0.009	0.191***	0.048	0.009	0.177***
Parent attitudes to teen gambling							0.055	0.038	0.042	0.027	0.037	0.020
Parental facilitation of gambling							0.715	0.059	0.431***	0.470	0.068	0.283***
	*R*^2^ = 0.056, *F*(3, 812) = 17.093, *p* < .001			*R*^2^ = 0.352, *F*(2, 813) = 221.152, *p* < .001			*R*^2^ = 0.457, *F*(10, 805) = 67.874, *p* < .001			*R*^2^ = 0.499, *F*(15, 800) = 53.042, *p* < .001		

Note. * *p* < .05, ** *p* < .01, *** *p* < .001

**Table 6:** Results of linear regression analyses with fathers (n = 367) examining predictors of adolescent gambling problems

	Model 1: Parent sociodemographic factors			Model 2: Adolescent factors			Model 3: Parental influences			Model 4: All predictors simultaneously		
	B	SE	β	B	SE	β	B	SE	β	B	SE	β
Parent age	-0.048	0.017	-0.145**							0.001	0.010	0.002
Indigenous status (ref: not)	1.820	0.425	0.216***							0.306	0.259	0.036
Parent education	0.391	0.115	0.168***							0.038	0.070	0.016
Adolescent gender (ref: male)				− 0.184	0.173	− 0.037				0.052	0.146	0.011
Adolescent impulsivity				− 0.020	0.022	− 0.035				− 0.014	0.020	− 0.024
Adolescent mental health problems				0.534	0.083	0.272***				0.080	0.084	0.040
Adolescent simulated gambling				0.943	0.062	0.597***				0.285	0.073	0.180***
Positive parenting							0.101	0.041	.093^a^	0.085	0.041	.078^a^
Inconsistent discipline							0.004	0.036	0.004	− 0.005	0.038	− 0.006
Poor supervision							0.012	0.036	0.015	0.006	0.036	0.008
Online restrictions (monitoring)							0.065	0.087	0.027	0.071	0.086	0.030
Online conversations (monitoring)							0.190	0.102	0.066	0.142	0.104	0.049
Parent-adolescent connectedness							− 0.415	0.094	− 0.166***	− 0.360	0.095	− 0.144***
Parent-adolescent hostility							0.100	0.091	0.043	0.110	0.092	0.047
Parent problem gambling							0.095	0.017	0.249***	0.084	0.017	0.219***
Parent attitudes to teen gambling							0.127	0.082	0.057	0.110	0.081	0.049
Parental facilitation of gambling							0.925	0.087	0.510***	0.693	0.099	0.382***
	*R*^2^ = 0.107, *F*(3, 363) = 14.515, *p* < .001			*R*^2^ = 0.573, *F*(4, 361) = 121.301 *p* < .001			*R*^2^ = 0.698, *F*(10, 356) = 82.385, *p* < .001			*R*^2^ = 0.717, *F*(17, 352) = 51.812, *p* < .001		

Note. a. This variable had a significant beta weight at *p* < .05. However, the relationship was negative in the bivariate analysis but has become positive in this multivariate analysis. This is a sign of a negative suppression effect of this variable and means that the association between positive parenting and gambling problems is difficult to interpret (Pandey & Elliott, 2010). Thus, it has not been discussed as an independent predictor in the presentation of results of this analysis

* *p* < .05, ** *p* < .01, *** *p* < .001

The fourth and final model considered all factors simultaneously. The analysis of mother data indicated that parental education, adolescent mental health problems, simulated gambling participation, poor parental supervision, parent-adolescent connectedness, parent problem gambling, and parental facilitation for gambling each independently predicted adolescent gambling problems. Independent predictors in the analysis on father data were simulated gambling participation, parent-adolescent connectedness, parent problem gambling, and parental facilitation of gambling. The strongest predictor in both analyses was parental facilitation of gambling.

## Discussion

This study has contributed to the limited research examining parental influences on adolescent gambling from the perspective of parents, and has provided updated information on the role of parents taking account of adolescent involvement in newer forms of gambling and simulated gambling. While prior research emphasised parental problem gambling as a primary intergenerational risk factor (Dowling et al. 2017a; McComb and Sabiston 2010), the present findings shift attention toward specific modifiable parenting behaviours that occur even in non-problem gambling parents. This study examined a range of parental influences—both gambling-related and related to the broader parenting context—on adolescent gambling. While several gambling-related parental influences and broader parenting factors were related to adolescent gambling, the strongest independent predictor of adolescent gambling participation and gambling problems was parental facilitation of adolescent gambling. Overall, this research highlights important parental risk and protective factors that can be targeted in population health initiatives aimed at protecting adolescents from problem gambling and gambling harm.

The first hypothesis of this study focused on the influence of gambling-related parenting behavior and attitudes on adolescent gambling. Consistent with the hypothesis, parent problem gambling, positive attitudes to adolescent gambling and parental facilitation of gambling were all significantly related at the bivariate level to greater adolescent gambling participation and gambling problems in both the mother and father samples. The extent to which parents facilitate their adolescent’s gambling was the strongest predictor of adolescent gambling. This was the case for both mothers and fathers, and after controlling for a comprehensive set of demographic and adolescent factors previously implicated in gambling behavior (Browne et al. 2019a, b; Dowling et al. 2017a). Other gambling-related parental factors also predicted independent variance in adolescent gambling outcomes, with maternal positive attitudes to adolescent gambling independently related to adolescent gambling participation, and maternal and paternal problem gambling independently related to adolescent gambling problems. However, the effect of parental gambling facilitation was independent of—and stronger than—these other gambling-related parental influences, indicating that what parents do with their adolescents related to gambling may be more influential than parental gambling characteristics alone.

These findings suggest that parental facilitative behaviors, such as allowing or encouraging gambling or participating in gambling with adolescents, may be more salient to adolescents than parents’ private attitudes or own gambling behavior, suggesting that parental facilitation is a key proximal mechanism of influence. Prior literature on parental influences on adolescent gambling development has often focused on parental problem gambling as a key risk factor leading to the intergenerational transmission of gambling behavior (Dowling et al. 2017a; McComb and Sabiston 2010). Studies with adolescents (Freund et al. 2023; Hing et al. 2021b) and findings from research with Canadian parents (Campbell et al., 2011) indicate that many parents endorse and are actively involved in their adolescent’s gambling. The current findings extend this body of research by identifying that there are gambling-related behaviors that many parents—both with and without a gambling problem—engage in that may place their adolescent child at risk of early participation in gambling and gambling problems. The findings indicate that among adolescents whose parents gamble, adolescents observe their parents’ gambling and are also supported or encouraged to gamble themselves in their parents’ presence, which likely conveys the message to adolescents that gambling is an acceptable and harmless activity. Thus, the strong direct effect of parental gambling facilitation speaks to the major role that parents likely play in condoning (or conversely, potentially preventing) their adolescent’s gambling behavior and may be a key mechanism for explaining the transmission of problem gambling from parents to their children. Given that early participation in gambling is a major risk factor for later problem gambling and gambling-related harm (Browne et al. 2019a, b), this is a significant concern and warrants further research and public health attention.

Consistent with the second hypothesis, almost all broader parenting influences were related to adolescent gambling for mothers and fathers. Lower levels of positive parenting and parent-adolescent connectedness and higher levels of inconsistent discipline, poor monitoring, and parent-adolescent hostility were associated with adolescent gambling at the bivariate level. Several of these factors were independent predictors in multivariate analyses. Poor maternal monitoring was associated with gambling participation and gambling problems, corroborating prior work with adolescents (Canale et al., 2017; Lee et al., 2014; Molinaro et al., 2014; Vachon et al., 2004). In contrast, online-specific monitoring did not uniquely predict adolescent gambling, which is consistent with findings from surveys of adolescents (Floros et al., 2013; King & Delfabbro, 2016). Given the current study was interested in adolescent gambling behavior in general and not online gambling behavior specifically, it makes sense that mothers with poorer awareness and supervision of their adolescent’s whereabouts and activities in general led to opportunities for their adolescent to engage in gambling. This finding also reinforces evidence that general parental monitoring exerts a stronger protective influence than technology-focused monitoring in preventing excessive internet use in adolescents (Vaala & Bleakley, 2015).

The quality of the parent-adolescent relationship was also uniquely related to adolescent gambling. Higher connectedness was negatively associated with adolescent gambling problems for mothers and fathers, while fathers’ positive parenting and relationship hostility were related to adolescent gambling participation. These findings suggest that connected, encouraging, and supportive relationships that are low in hostility and criticism may protect against adolescent gambling, supporting previous research with adolescents highlighting the protective role of positive parent-adolescent relationships (Floros et al., 2013; Molinaro et al., 2014; Pisarska & Ostaszewski, 2020). Although this study suggests that parental facilitation of gambling is a proximal influence, it is possible that broader parenting processes, such as effective monitoring and positive parent-adolescent relationships, function as contextual moderators, shaping the extent to which gambling-specific parental behaviours translate into adolescent gambling outcomes. Prior work indicates that strong parent–adolescent bonds buffer adolescents from the negative effects of parental gambling (Dowling et al. 2017b) and exposure to gambling advertising (Parrado-González & León-Jariego, 2020), underscoring the need for longitudinal research to clarify how these protective mechanisms operate. Collectively, the study’s findings indicate that the broader parenting context appears to play a protective role in limiting adolescent gambling.

Another important finding from this study was that adolescent simulated gambling participation was an independent and comparably strong predictor of mother-reported adolescent gambling participation and gambling problems, and of father-reported adolescent gambling problems. Research with adolescents suggests that young people who engage in simulated gambling are more likely to gamble on monetary forms of gambling and to experience gambling problems (Hing et al., 2022 a; Hayer et al., 2018). The current study extends this work by showing that these associations are present using parent-reported information, and adds weight to calls for longitudinal research to better understand if there is a pathway from simulated to monetary gambling (Delfabbro & King, 2021) and the role that parents might play in either disrupting or facilitating that trajectory.

Finally, other independent predictors of adolescent gambling included maternal Indigeneity (with gambling participation) and maternal education (with gambling problems), although the effects of these factors were minimal once adolescent and parent influences were considered. Conversely, other parental demographic (e.g., age, income, family structure, English as primary language) and adolescent factors (i.e., age, gender, impulsivity) were not independently related to adolescent gambling. This indicates that more proximal factors, particularly parental gambling facilitation, have a more direct impact on the gambling behavior of adolescents. Importantly, because gambling-related and general parenting influences are modifiable through intervention, these should serve as better intervention targets for addressing adolescent gambling and gambling harm.

### Strengths, Limitations and Future Directions

The use of online panels for data collection provided a large sample that included many fathers (31.0%) as well as good representation of single parents (19.7%), families with household incomes below the national median (38.0%), and Indigenous parents (7.3%). The relatively large number of fathers recruited for this study is an important strength, given that fathers tend to be poorly represented in research on parenting (Fabiano & Caserta, 2018). However, the online panel sampling method means that the sample was not representative of the Australian parenting population. Another limitation was the cross-sectional research design, which means that firm conclusions about the causal influence of parental behavior on adolescent gambling cannot be made. Further, although the lack of parental data on adolescent gambling was an important gap that this study helps to address, relying solely on parent-report data means the findings reflect only parental perceptions of their own behaviors and attitudes and of their adolescent’s gambling. These limitations could be addressed in future by using prospective longitudinal designs drawing on triadic data from mothers, fathers and adolescents.

Finally, it is important to acknowledge that several of the measures were purpose-built for this survey and/or adapted from prior work (e.g., parental online monitoring, parental gambling attitudes). Thus, we need to interpret the findings cautiously because these measures have not been subject to formal psychometric evaluation. This is particularly the case for the measure used to assess adolescent gambling problems (i.e., DSM-IV-MR-J), which was originally developed and validated as a self-report measure for adolescents. Because a parent-report measure of adolescent problems was not available, we adapted the wording of the items and included an “I don’t know” option on the rating scale so it was suitable for parents to report on their adolescent’s gambling behavior. This means that findings related to adolescent problem gambling need to be interpreted cautiously given that this measure has not been validated for parent use. Given that parent-report questionnaires are routinely used in research and clinical settings for adolescent behavioral and emotional concerns (De Los Reyes et al., 2015) and have been found to more reliably predict adolescent psychiatric diagnoses compared to adolescent reports (Kuhn et al., 2017), there is a clearly a need to develop and validate a parent-report measure of adolescent gambling problems. This would enable future research to draw on both parent and adolescent reports to provide a more comprehensive understanding of adolescent gambling.

### Conclusions

This study highlighted the role of parents as potential enablers or inhibitors of adolescent gambling. Parental facilitation of gambling was identified as a likely risk factor for adolescent gambling, while effective monitoring and positive parent-adolescent relationships may serve as important protective factors. Conceptually, the findings support bioecological perspectives on adolescent gambling by highlighting the importance of considering modifiable family-level processes alongside individual characteristics in explanatory models of problem gambling in adolescents (Savard et al., 2015). While further longitudinal research is needed to confirm the influence of these parental factors on adolescent gambling, the current findings suggest that parental behavior should serve as an intervention target for prevention and intervention efforts. Given the pervasive influence of gambling advertising, potentially accessible online gambling, and probable risks of simulated gambling (Noble et al., 2022), public health initiatives should focus on supporting parents to understand and manage the potentially detrimental influence of the contemporary gambling landscape on their adolescents. This will be particularly important—but likely more challenging—in countries like Australia, where gambling is embedded within the national culture as a socially acceptable and enjoyable pastime (Marko et al., 2022). Nonetheless, these findings highlight the central role of parents in shaping adolescent gambling outcomes and point to actionable strategies for reducing gambling-related harm among young people.

## Supplementary Information

Below is the link to the electronic supplementary material.

Supplementary Material 1 (DOCX 34.3 KB)

## Data Availability

Data associated with this project is stored securely by CQUniversity, Australia. Requests for access to the data can be made in writing to the corresponding author.

## Footnotes

1 Skins are digital items in online games that change the appearance, and sometimes function, of weapons or characters. Skins betting is a form of online gambling where people use skins as a currency to bet on esports, games of chance, or other events, often through third-party websites outside the game environment. These skins can acquire real-world monetary value and are used similarly to cash in gambling contexts (Hing et al. 2021a).

2 Two parents reported that they were < 15 years older than their adolescent: one 20-year-old parent who indicated they were an older sibling in a guardian role, and one 26-year-old who indicated they were a stepparent. Because both participants reported they were involved in a parental or caregiver role with their nominated adolescent, they were included in the data set.
