# Supplementary material for: The Role of Parents in Adolescent Gambling Participation and Gambling Problems
Source: J Gambl Stud. 2026 Jun 4;42(3):993–1019. doi: 10.1007/s10899-026-10520-4 (PMC13577966; doi:10.1007/s10899-026-10520-4)
Supplement: Supplementary file 1 [file 10899_2026_10520_MOESM1_ESM.docx]

**Supplementary Material 1**

*Frequency of Engagement by Fathers in Behaviors that may Facilitate Adolescent Gambling (Ordered from Most Frequent to Least Frequent)*

Proportion of Participants

| Parental Behavior | More than once a week | About once a week | 2-3 times per month | About once a month | Less than once a month | Never in the last 12 months |
| --- | --- | --- | --- | --- | --- | --- |
| Allowed teen to use your online gambling account to gamble | 1.6 | 6.3 | 5.4 | 5.4 | 4.9 | 76.3 |
| Allowed teen access to online gambling activities on your account | 2.7 | 4.6 | 4.6 | 6 | 6.5 | 75.5 |
| Given teen money to gamble | 4 | 5.2 | 3.5 | 5.4 | 7.1 | 74.7 |
| Allowed teen to use own money to gamble | 3.8 | 3.3 | 6.3 | 4.9 | 7.9 | 73.8 |
| Included teen in private betting with money (e.g., sweepstakes, footy tipping) | 4.7 | 5.2 | 3.3 | 5.2 | 9 | 72.8 |
| Put a bet on for teen for a sports, esports, racing or novelty event | 4.6 | 4.6 | 4.4 | 5.7 | 8.7 | 71.9 |
| Taken teen to gambling venue | 3.2 | 3.5 | 6.3 | 4.1 | 12 | 70.8 |
| Involved teen in betting on sports or esports | 4.6 | 4.9 | 6.3 | 4.4 | 9.5 | 70.3 |
| Played cards, board games, or other games at home for money | 3.2 | 4.1 | 6 | 6.5 | 10.1 | 70 |
| Talked about gambling as something fun or exciting to do | 3.3 | 5.4 | 4.9 | 6 | 10.6 | 69.8 |
| Bought scratch tickets or lottery tickets for teen | 3.3 | 5.2 | 6.5 | 7.4 | 10.4 | 67.3 |
| Agreed on rules about teen gambling | 4.1 | 5.2 | 6 | 6.3 | 13.4 | 65.1 |
| Asked teen to pick lucky numbers in keno or lottery | 3.8 | 3.8 | 7.1 | 10.4 | 11.4 | 63.5 |
| Talked about your own gambling wins with teen | 3.3 | 6.3 | 5.7 | 7.6 | 15.5 | 61.6 |

*Frequency of Engagement by Mothers in Behaviors That May Facilitate Adolescent Gambling (Ordered from Most Frequent to Least Frequent)*

Proportion of Participants

| Parental Behavior | More than once a week | About once a week | 2-3 times per month | About once a month | Less than once a month | Never in the last 12 months |
| --- | --- | --- | --- | --- | --- | --- |
| Allowed teen to use your online gambling account to gamble | 0.9 | 0.7 | 1.3 | 1.6 | 2.2 | 93.3 |
| Allowed teen access to online gambling activities on your account | 0.4 | 0.9 | 0.9 | 1.5 | 3.3 | 93.1 |
| Given teen money to gamble | 0.9 | 0.6 | 1.2 | 1.2 | 3.7 | 92.4 |
| Allowed teen to use own money to gamble | 1.2 | 1 | 0.7 | 1.2 | 3.7 | 92.2 |
| Played cards, board games, or other games at home for money | 0.6 | 0.7 | 1.6 | 2.8 | 4.7 | 89.6 |
| Put a bet on for teen for a sports, esports, racing or novelty event | 0.7 | 0.7 | 2.2 | 1.3 | 5.8 | 89.2 |
| Talked about gambling as something fun or exciting to do | 0.7 | 1.1 | 1 | 2.5 | 5.8 | 89 |
| Involved teen in betting on sports or esports | 0.6 | 1 | 1.5 | 1.5 | 7.1 | 88.4 |
| Included teen in private betting with money (e.g., sweepstakes, footy tipping) | 0.5 | 0.5 | 1.3 | 1.6 | 8.3 | 87.7 |
| Agreed on rules about teen gambling | 0.9 | 1.2 | 1.1 | 3.9 | 8.3 | 84.6 |
| Taken teen to gambling venue (e.g., racetrack, poker machine area in pub/club) | 0.5 | 0 | 2.3 | 2.8 | 9.9 | 84.4 |
| Bought scratch tickets or lottery tickets for teen | 0.6 | 1.5 | 2.1 | 2.9 | 10.3 | 82.6 |
| Asked teen to pick lucky numbers in keno or lottery | 0.8 | 0.7 | 2.9 | 4.7 | 14.8 | 76 |
| Talked about your own gambling wins with teen | 0.7 | 1.8 | 1.3 | 4.4 | 15.8 | 75.9 |

**Supplementary Material 2**

*Bivariate Relationships Among Adolescent Factors, Parental Factors and Adolescent Gambling for Mothers (Upper Diagonal) and Fathers (Lower Diagonal)*

|  | 1 | 2 | 3 | 4 | 5 | 6 | 7 | 8 | 9 | 10 | 11 | 12 | 13 | 14 | 15 |
| --- | --- | --- | --- | --- | --- | --- | --- | --- | --- | --- | --- | --- | --- | --- | --- |
| 1. Adolescent impulsivity |  | .349^***^ | .085^*^ | -.216^***^ | .318^***^ | .319^***^ | -.019 | -.174^***^ | -.208^***^ | .406^***^ | .097^**^ | .037 | .029 | .025 | .040 |
| 2. Adolescent mental health | .402^***^ |  | .186^***^ | -.135^***^ | .260^***^ | .258^***^ | .059 | -.073^*^ | -.131^***^ | .313^***^ | .237^***^ | .149^***^ | .225^***^ | .160^***^ | .252^***^ |
| 3. Adolescent simulated gambling | .105^*^ | .451^***^ |  | -.224^***^ | .174^***^ | .297^**^ | .027 | -.101^**^ | -.233^***^ | .210^***^ | .461^***^ | .374^***^ | .696^***^ | .540^***^ | .575^***^ |
| 4. Positive parenting | -.241^***^ | -.182^***^ | -.167^***^ |  | -.203^***^ | -.266^***^ | .063 | .371^***^ | .590^***^ | -.271^***^ | -.203^***^ | -.188^***^ | -.240^***^ | -.187^***^ | -.235^***^ |
| 5. Inconsistent discipline | .358^***^ | .539^***^ | .362^***^ | -.045 |  | .385^***^ | .056 | -.122^***^ | -.253^***^ | .404^***^ | .256^***^ | .175^***^ | .215^***^ | .179^***^ | .205^***^ |
| 6. Poor supervision | .246^***^ | .510^***^ | .471^***^ | -.278^***^ | .605^***^ |  | -.073^*^ | -.253^***^ | -.362^***^ | .378^***^ | .330^***^ | .281^***^ | .368^***^ | .317^***^ | .375^***^ |
| 7. Online restrictions (monitoring) | .086 | .329^***^ | .274^***^ | .112^*^ | .314^***^ | .242^***^ |  | .375^***^ | .116^**^ | .128^***^ | .030 | -.032 | .108^**^ | .076^*^ | .089^*^ |
| 8. Online conversations (monitoring) | -.022 | .279^***^ | .156^**^ | .270^***^ | .198^**^ | .171^***^ | .507^***^ |  | .475^***^ | -.147^**^ | -.131^***^ | -.129^***^ | -.138^***^ | -.078^*^ | -.128^***^ |
| 9. Parent-adolescent connectedness | -.226^***^ | -.207^***^ | -.263^***^ | .599^***^ | -.037 | -.263^***^ | .132^*^ | .266^***^ |  | -.274^***^ | -.238^***^ | -.241^***^ | -.251^***^ | -.209^***^ | -.282^***^ |
| 10. Parent-adolescent hostility | .304^***^ | .552^***^ | .398^***^ | -.265^***^ | .495^***^ | .584^***^ | .225^***^ | .073 | -.225^***^ |  | .258^***^ | .125^***^ | .278^***^ | .193^***^ | .239^***^ |
| 11. Parent problem gambling | .180^***^ | .517^***^ | .644^***^ | -.254^***^ | .410^***^ | .521^***^ | .272^***^ | .116^*^ | -.245^***^ | .508^***^ |  | .279^***^ | .608^***^ | .428^***^ | .522^***^ |
| 12. Parent attitudes to adolescent gambling | .098 | .356^***^ | .508^***^ | -.193^***^ | .249^***^ | .408^***^ | .138^**^ | .086 | -.202^***^ | .371^***^ | .530^***^ |  | .415^***^ | .356^***^ | .325^***^ |
| 13. Parent facilitation of adolescent gambling | .157^**^ | .551^***^ | .758^***^ | -.196^***^ | .411^***^ | .553^***^ | .350^***^ | .187^***^ | -.235^***^ | .493^***^ | .728^***^ | .572^***^ |  | .657^***^ | .633^***^ |
| 14. Adolescent gambling participation | .107^*^ | .415^***^ | .590^***^ | -.248^***^ | .311^***^ | .461^***^ | .264^***^ | .150^**^ | -.218^***^ | .451^***^ | .567^***^ | .444^***^ | .716^***^ |  | .575^***^ |
| 15. Adolescent gambling problems | .142^**^ | .526^***^ | .720^***^ | -.175^***^ | .384^***^ | .513^***^ | .318^***^ | .196^***^ | -.295^***^ | .476^***^ | .714^***^ | .529^***^ | .797^***^ | .625^***^ |  |

*Note.* * *p* < .05, ** *p* < .01, *** *p* < .001
